# Clinical features of patients with paroxysmal kinesigenic dyskinesia, mutation screening of *PRRT2* and the effects of morning draughts of oxcarbazepine

**DOI:** 10.1186/s12887-019-1798-7

**Published:** 2019-11-14

**Authors:** Gang Pan, Linmei Zhang, Shuizhen Zhou

**Affiliations:** Children’s Hospital Of Fudan University, 399 Wan Yuan Road, Shanghai, Minhang District China

**Keywords:** Clinical features, OXC, PKD, *PRRT2*, Treatment

## Abstract

**Background:**

The objective of this study was to summarize clinical features and *PRRT2* mutations of paediatric paroxysmal kinesigenic dyskinesia (PKD) patients and observe the tolerability and effects of morning draughts of oxcarbazepine.

**Methods:**

Twenty patients diagnosed with PKD at Children’s Hospital of Fudan University between January 2011 and December 2015 were enrolled. These patients’ medical records were reviewed. Peripheral venous blood was obtained from all enrolled patients, and polymerase chain reaction (PCR) and Sanger sequencing were used to sequence proline-rich transmembrane protein 2 (*PRRT2*) gene mutations. Clinical features of PKD patients with and without *PRRT2* mutations were compared. All enrolled patients were treated with morning draughts of oxcarbazepine (OXC). The starting dose was 5 mg/kg·d, and the dose was increased by 5 mg/kg·d each week until attacks stopped. Effective doses and adverse effects were recorded.

**Results:**

For all enrolled patients, dyskinesia was triggered by sudden movement. Dyskinetic movement usually involved the limbs and was bilateral; the majority of enrolled patients exhibited both dystonia and choreoathetosis. We identified *PRRT2* mutations in 5 patients, including 4 familial patients and 1 sporadic patient. All 20 patients took low doses of OXC (5–20 mg/kg·d) as draughts in the morning, and dyskinesia attacks stopped in 19 patients.

**Conclusions:**

Paediatric PKD patients have various phenotypes. *PRRT2* mutations are common in familial cases. OXC taken as morning draughts can be a treatment option for paediatric PKD patients.

## Introduction

Paroxysmal kinesigenic dyskinesia (PKD) (OMIM 128200) is the most common form of paroxysmal dyskinesia. The main clinical feature of PKD is brief, unilateral or bilateral attacks of dystonia and choreoathetosis triggered by sudden movements [1]. The prevalence of PKD is ~ 1/150000, certain patients have a family history of PKD, which is typically inherited in an autosomal dominant pattern [2]. The onset age for PKD is typically in childhood or adolescence, and symptoms usually become self-limiting with age [3]. Recently, two Chinese groups had identified mutations in the gene encoding proline-rich transmembrane protein 2 (*PRRT2*) (OMIM 614386) as a cause of PKD, the most common mutation in *PRRT2* was c.649dupC(p.R217Pfs*8) [4, 5].

The pathogenesis and mechanism of PKD have not been completely elucidated. Recently, researchers have found abnormal excitability in the cortex and basal ganglia in certain PKD patients and concluded that channelopathy is an underlying mechanism in PKD [6]. Most PKD patients exhibit a favourable response to carbamazepine (CBZ), which modulates different types of calcium channels, supporting the hypotheses that channelopathy is an underlying mechanism in PKD [7]. However, allergies are present in a subset of PKD patients who use CBZ; thus, a safer and efficient alternative drug must be identified. Oxcarbazepine (OXC), a derivative of CBZ, has been reported to be effective in certain cases of PKD [8].

Few available reports address treatment for paediatric PKD patients. This research sought to investigate clinical manifestations and genetic characteristics of *PRRT2* mutations and the correlation between PKD phenotype and the presence of mutations in the *PRRT2*; in addition, the effects of morning draughts of OXC on paediatric PKD patients were observed.

## Methods

### Subjects

Twenty Chinese-Han patients with PKD at Children’s Hospital of Fudan University were enrolled between January 2011 and December 2015. All subjects satisfied the following diagnostic criteria for PKD: an identified trigger for attacks (sudden movements), short attack durations (< 1 min), a lack of loss of consciousness or pain during attacks, antiepileptic drug responsiveness, the exclusion of other organic diseases, and age at onset of 1 to 20 years. Disease histories, clinical manifestations, results from physical, neurological, and biochemical examinations, and electroencephalography (EEG) and neuroimaging findings were investigated and analysed.

### Genetic analysis

Genomic DNA was extracted from patients’ peripheral blood leukocytes. Polymerase chain reaction (PCR) and Sanger sequencing were performed to identify *PRRT2* mutations, and no attempt was made to screen other relevant genes implicated in dystonia like *MECR*, *SLC2A1*, *SGCE*, *THAP1*, *EKD2*, *PNKD2*, *DYT7*, CNVs were not assessed. Two coding exons (2 and 3) were amplified by PCR (Exon 2AF 5′-CTCCTCCTCTTCCAGGGTTT-3′, Exon 2AR 5′-TTTTTGAGGGTGGTGAGTGA-3′, Exon 2BF 5′-TCTGAGAGTGTAGGGGAAAAGC-3′, Exon 2BR 5′-CTAGGGAGAGGCAAACAAAGG-3′, Exon 3F 5′-TCCACCTGATCCCTTCTGG-3′ and Exon 3R 5′-CAGGCTCCCTTGGTCCTTAG-3′). We used touch down PCR, denature last 3 min at 94 °C, the first thermal cycling was 16 cycles of 94 °C for 30 s, 65 °C for 30 s and 72 °C for 40 s, the second thermal cycling was 30 cycles of 94 °C for 30 s, 60 °C for 30 s and 72 °C for 40 s. GRCh37 was utilized as reference genome and NM_145239.2 was utilized for mutation screening. Clinical features of PKD patients with and without *PRRT2* mutations were compared. Differences between two groups were compared using unpaired Student’s t-tests and Fisher’s exact test for quantitative and categorical variables, respectively. The threshold for statistical significance was *p* < 0.05.

### Treatment

OXC, which was taken as a draught in the morning, was used to treat all enrolled patients. The starting dose was 5 mg/kg·d. The frequency of attacks and adverse events were recorded by at least two caregivers of each patient. The dose was increased by 5 mg/kg·d each week until both two caregivers recorded an at least 75% reduction in attacks frequency or reached the maximum dose in the drug instruction. Only when the attack frequency decreased more than 75% last for 1 month, the dose would be recorded as effective dose.

## Results

### Clinical manifestations and *PRRT2* mutations of enrolled PKD patients

**Fig. 1:**
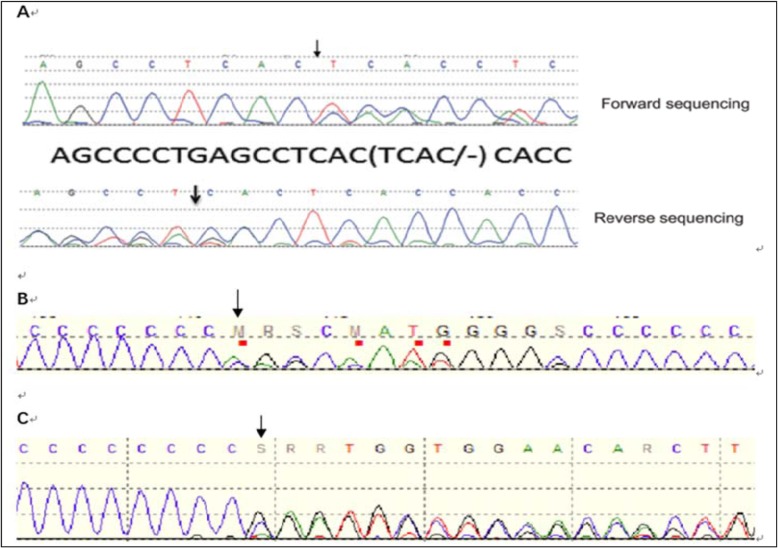
Chromatograms of the detected variants. **a** Sequence traces of familial PRRT2 mutation with forward and reverse sequences reveal the deletion of TCAC at position 604–607, **b** Sequence traces of PRRT2 mutation reveal the duplication of C at position 629, **c** Sequence traces of PRRT2 mutation reveal the duplication of C at position 649

Five familial PKD patients from 4 families (including two patients from one family whose cases have been described in a previously published report [9]) and 15 sporadic PKD patients were enrolled in our study (17 males and 3 females) (Table 1). The age of dyskinesia onset ranged from 2.5 to 13 years, and the mean and median ages were 8.1 and 8 years, respectively. All patients had normal results on physical, neurological, and biochemical examinations and normal EEG and neuroimaging findings. Paroxysmal dyskinesia was triggered by sudden movement in all enrolled patients. Dyskinetic attacks involved the limbs for all enrolled patients, two of them had focal onset in one limb; one patient had facial involvement. Eighteen patients had bilateral dyskinesia, and the remaining patients exhibited unilateral dyskinesia. The phenomenology of attacks was primarily dystonia in 10 patients, primarily choreoathetosis in 4 patients, and both dystonia and choreoathetosis in the remaining 6 patients. The frequency of attacks ranged from several times per day to several times per week. Infantile convulsions with paroxysmal choreoathetosis were observed in 3 familial PKD patients from 2 families. One sporadic case involved partial epilepsy, and the remaining cases involved no comorbidities. *PRRT2* mutations were identified in 4 of the 5 familial PKD patients, only point mutations or small indels were tested, in patient 1 and 2 from the same family we found c.604-607delTCAC(chr16:29824979–29,824,982, p.S202Hfs*25, the allele frequency was none), c.629dupC(chr16:29825004, p.Ala211Serfs*14, the allele frequency was 0.0001 in ExAC) and c.649dupC(chr16:29825024, p.Arg217Profs*8, the allele frequency was 0.0000 in ExAC) were found in patient 3 from family 2 and patient 5 from family 4, we also found c.649dupC(chr16:29825024, p.Arg217Profs*8, the allele frequency was 0.0000 in ExAC) in one sporadic PKD patients (Fig. 1).

**Table 1:** Patients clinical features

Patients	Age at onset (years)	Family	Phenomenolo-gy of attack	Localisation	PRRT2 mutations	Frequency of attack (per day)	Comorbidity
1	2.7	fam1	D + C	Upper and lower limbs	c.604-607delTCAC p.S202Hfs*25	< 5	ICCA
2	7.6	fam1	C	Lower limbs	c.604-607delTCAC p.S202Hfs*25	< 5	ICCA
3	7.8	fam2	C	Lower limbs	c.629dupC p.Ala211Serfs*14	5 to 10	ICCA
4	2.5	fam3	D	Lower limbs	–	> 10	
5	10	fam4	D	Upper and lower limbs	c.649dupC p.Arg217Profs*8	5 to< 10	
6	6.7	S	D	Lower limbs	–	< 5	
7	9.8	S	D	Right limbs	–	5 to< 10	
8	13	S	D + C	Upper and lower limbs	–	5 to< 10	
9	5.2	S	D	Left lower limb	–	< 5	
10	5	S	D	Lower limbs	–	< 5	
11	13	S	D	Right upper limb	–	< 5	
12	4.7	S	D + C	Upper and lower limbs facial	–	> 10	
13	13	S	D	Upper and lower limbs	–	5to < 10	
14	12.5	S	D	Lower limbs	–	< 5	
15	9.6	S	D + C	Lower limbs	–	> 10	
16	10.1	S	D	Right limbs	–	5 to< 10	
17	3	S	C	Upper limbs	–	< 5	Partial epilepsy
18	7.6	S	C	Upper limbs	–	5 to< 10	
19	8.2	S	D + C	Upper and lower limbs	c.649dupC p.Arg217Profs*8	5 to< 10	
20	10	S	D + C	Upper and lower limbs	–	5 to< 10	

*M* Male, *F* Female, *S* Sporadic, *D* Dystonia, *C* Choreoathetosis, *ICCA* Infantile convulsions with paroxysmal choreoathetosis

### Treatment effects

All enrolled patients took morning draughts of OXC, which stopped attacks in 19 patients. One sporadic patient was treated for 3 weeks but still had a < 75% reduction in attacks frequency after took the dose of 15 mg/kg·d for 1 week since the dose was increased by two times and refused further treatment. All the last 19 patients had a > 75% reduction in attacks frequency last more than 1 month, the effective dose was 5 mg/kg·d in 2 patients, 10 mg/kg·d in 13 patients, 15 mg/kg·d in 3 patients and 20 mg/kg·d in one patient. The median effective dose was 10 mg/kg·d. One patient experienced a slight rash that was alleviated with antiallergic medicines and none had serious idiosyncratic adverse effects.

## Discussion

### Clinical manifestations and genetic characteristics of PRRT2 mutations

The *PRRT2*, which encodes proline-rich transmembrane protein 2, is composed of four exons; this protein consists of 340 amino acids and contains two predicted transmembrane domains [10]. The *PRRT2* is expressed primarily in the brain, particularly in the cerebral cortex and basal ganglia; this expression pattern may explain why abnormal excitability in the cortex and basal ganglia is detected in a subset of PKD patients [6]. However, the exact function of the *PRRT2* remains unknown. Reports have indicated that this gene interacts with synaptosomal-associated protein 25 kDa (SNAP25) [11]. SNAP25 is a presynaptic plasma-membrane-bound protein involved in the synaptic vesicle membrane docking and fusion pathway and plays a key role in calcium-triggered neuronal exocytosis [12]. Its binding partner PRRT2 may also play a role in this process [13]. The absence or low expression of PRRT2 due to mutations in the *PRRT2* may result from the loss of a transmembrane domain, which renders PRRT2 unable to anchor to membranes; such changes in PRRT2 may lead to hyperexcitability and trigger dyskinesias [13]. However, further investigation is needed to elucidate how *PRRT2* mutations cause PKD.

*PRRT2* mutations are present in most familial PKD patients; the most common such mutation is c.649dupC [10]. Among sporadic patients, the rate of *PRRT2* mutations ranges from 20 to 45%, with c.649delC and c.649dupC as commonly observed mutations [14]. In addition to PKD patients, *PRRT2* mutations have also been identified in patients with infantile convulsions with paroxysmal choreoathetosis, benign familial infantile epilepsy [15], migraine or episodic ataxia [16]. This phenomenon could explain why certain PKD patients have comorbidities such as migraine or seizures [17]. Researchers have determined that among PKD patients, younger age at symptom onset, good response to CBZ and predisposition to non-dyskinetic symptoms are significantly correlated with *PRRT2* mutations [18, 19].

We enrolled a total of 20 patients. *PRRT2* mutations were detected in 5 patients but not the remaining 15 patients. Four of the 5 patients positive for *PRRT2* mutations had familial PKD. Differences in *PRRT2* mutations between familial PKD patients and sporadic PKD patients were evaluated using unpaired Student’s t-tests or Fisher’s exact test (Table 2). We found a significantly higher positive rate of *PRRT2* mutations among familial PKD patients than among sporadic PKD patients (*p* < 0.05); similarly, prior studies revealed that *PRRT2* mutations were related to PKD, especially familial PKD [10]. PKD patients with *PRRT2* mutations had more comorbidities (*p* < 0.05). Gender distribution and the clinical phenotypes of PKD did not differ between PKD patients with *PRRT2* mutations and PKD patients without such mutations. Although many studies have reported that PKD patients with *PRRT2* mutations are younger at disease onset than other PKD patients, in our study, there were no differences between these two patient groups. This finding may be attributable to the small sample sizes in our study or to differences among different populations.

**Table 2:** Comparison of clinical features between the PKD patients with and without *PRRT2* mutations

	patients with PRRT2 mutations	patients without PRRT2 mutations	*p*
No. of subjects	5	15	
Male(%)	4(80%)	13(86.7%)	1.000
Age at onset (years)			0.502
Mean (SD)	7.1(3)	8.4(3.7)	
Median	7	9	
Main phenotype, n			0.338
Choreoathetosis	2	2	
Dystonia	1	9	
Mixed	2	4	
Laterality of dyskinesia			0.530
Unilateral	0	4	
Biateral	5	11	
Involved limb			0.805
Upper limbs	0	3	
Lower limbs	2	6	
Both	3	6	
Frequency of attack/day, n			0.805
< 5	2	6	
5–10	3	6	
> 10	0	3	
Comorbidity	3	1	0.032

P^*^ < 0.05

### Treatment for PKD

The pathogenesis of PKD has not yet been elucidated; however, recent evidence of abnormal excitability in the cortex and basal ganglia in PKD patients supports the hypothesis that channelopathy is an underlying mechanism of PKD [20]. Moreover, certain studies report that PKD patients usually exhibit good response to antiepileptic drugs, including lamotrigine, phenytoin, valproic acid, CBZ, and OXC, all of which achieve treatment objectives through the mechanism of modulating ion channels [21].

Prior studies have suggested that a low dosage of CBZ (75–300 mg/d) can significantly improve paroxysmal dyskinesia for certain PKD patients during the first week of treatment; however, attacks will recur once the medication is withdrawn or missed [22]. Although CBZ benefits certain PKD patients, the adverse effects, which include serious idiosyncratic adverse effects, cannot be ignored. Toxic epidermal necrolysis and Stevens-Johnson syndrome are serious dermatological reactions to CBZ. The incidence of these reactions is 1 in 60,000 among Caucasians, but the risk is tenfold higher in certain Asian countries due to HLA-B1502 [23]. As a result, the use of CBZ is limited in various Asian countries, including China. As a structural derivative of CBZ, OXC mainly blocks voltage-sensitive sodium currents [24]. Unlike CBZ, OXC is not metabolized by the hepatic cytochrome-P450 enzyme system, and OXC has a lower plasma protein binding rate than CBZ; thus, OXC has fewer side effects and drug interactions [25]. OXC is rapidly and extensively reduced by cytosolic hepatic enzymes to its monohydroxylated derivative (MHD), which represents the antiepileptic agent and the active metabolite of OXC [26]. Half-lives in a healthy person are 1–5 h for OXC and 7–20 h for MHD [24]. A retrospective study addressing the use of CBZ or OXC monotherapy for PKD from 2005 to 2011 found that both CBZ and OXC had similar efficacies and tolerability. Patients experienced significant improvement with low doses of OXC (75–300 mg/d) or CBZ (50–300 mg/d), but 3 patients exhibited better tolerance after conversion from CBZ to OXC [8].

Because attacks of dystonia and choreoathetosis are triggered by sudden movements and patients may engage in more movements during the day, attacks are more likely to occur during the day than at night. Given this characteristic of PKD and the half-lives of OXC and MHD, OXC taken as a morning draught may have good efficacy and improve quality of life. This study observed the efficacy of low doses of OXC taken as a draught in the treatment of paediatric PKD patients. Prior reports have already showed that low doses of OXC are effective for PKD patients. Moreover, because attacks associated with PKD were mainly triggered by movement during the day, to decrease the frequency of drug administration and improve therapy compliance, all 20 enrolled patients took OXC as morning draughts. All but 1 of the 20 patients exhibited considerable improvement in dystonia or choreoathetosis with low doses of OXC (5–20 mg/kg·d).

### Differential diagnosis and misdiagnosis of PKD patients

PKD should be distinguished from the other paroxysmal dyskineias such as paroxysmal non-kinesigenic dyskinesia (PNKD) (OMIM 118800) and paroxysmal exertion-induced dyskinesia (PED) (OMIM 612126) as the similar movement disorder could present in all of them [1]. PED need physical exercise for more long time than PKD to trigger and last time varies from 5 to 30 min [7].Unlike PKD and PED, the cause of PNKD has nothing to do with voluntary movement or exercise, attack could be triggered by caffeine, alcohol, excitement and fatigue, the symptom duration minutes to 4 h.

As a paroxysmal disease, PKD can also be misdiagnosed as epilepsy, especially partial epilepsy, given that PKD patients are conscious during dyskinesia attacks. After treatment with antiepileptic drugs, certain PKD patients may not experience attacks, which may lead doctors to mistakenly believe that epilepsy is a reliable diagnosis for these patients. Moreover, PKD patients may have seizures as a comorbidity, contributing to misdiagnoses of PKD.

Two-thirds of the patients in our group had been misdiagnosed with epilepsy; therefore, it is necessary for doctors to distinguish between PKD and epilepsy to improve diagnostic accuracy. Attacks of dyskinesia in PKD patients are triggered by sudden movements, which may help distinguish PKD from epilepsy. Video-Electroencephalogram (VEEG), which can reveal whether epileptic discharge is present during an attack, could be a major approach for the diagnosis and differential diagnosis of PKD. It is also important to identify a syndrome when PKD patients experience seizure attacks, such as infantile convulsions with paroxysmal choreoathetosis (ICCA) (OMIM 602066) or benign familial infantile epilepsy (OMIM 605751).

## Conclusion

This study focuses on the clinical features and treatment of paediatric PKD patients. As a rare and paroxysmal disease, PKD can be misdiagnosed; therefore, it is necessary to enhance doctors’ knowledge of PKD and reduce the rate of erroneous diagnosis. Paediatric PKD patients have various phenotypes. *PRRT2* mutations are common in familial cases, whereas additional study of the genetic mechanisms of PKD in sporadic cases is required. Attacks in paediatric PKD patients can be improved by OXC taken as morning draughts.

## Data Availability

All data generated or analysed during this study are included in this published article.

## Abbreviations

CBZ: Carbamazepine
ICCA: Infantile convulsions with paroxysmal choreoathetosis
MHD: Monohydroxylated derivative
OXC: Oxcarbazepine
PCR: Polymerase chain reaction
PED: Paroxysmal exertion-induced dyskinesia
PKD: Paroxysmal kinesigenic dyskinesia
PNKD: Paroxysmal non-kinesigenic dyskinesia
PRRT2: Proline-rich transmembrane protein 2
SNAP25: Synaptosomal-associated protein 25 kDa
VEEG: Video-Electroencephalogram
